# Glycosyltransferase-Like RSE1 Negatively Regulates Leaf Senescence Through Salicylic Acid Signaling in *Arabidopsis*

**DOI:** 10.3389/fpls.2020.00551

**Published:** 2020-05-15

**Authors:** Seulbee Lee, Myung-Hee Kim, Jae Ho Lee, Jieun Jeon, June M. Kwak, Yun Ju Kim

**Affiliations:** ^1^Center for Plant Aging Research, Institute for Basic Science, Daegu, South Korea; ^2^Department of New Biology, Daegu Gyeongbuk Institute of Science and Technology, Daegu, South Korea

**Keywords:** cell wall, glycosylation, glycosyltransferase, leaf senescence, salicylic acid

## Abstract

Leaf senescence is a developmental process designed for nutrient recycling and relocation to maximize growth competence and reproductive capacity of plants. Thus, plants integrate developmental and environmental signals to precisely control senescence. To genetically dissect the complex regulatory mechanism underlying leaf senescence, we identified an early leaf senescence mutant, *rse1*. *RSE1* encodes a putative glycosyltransferase. Loss-of-function mutations in *RSE1* resulted in precocious leaf yellowing and up-regulation of senescence marker genes, indicating enhanced leaf senescence. Transcriptome analysis revealed that salicylic acid (SA) and defense signaling cascades were up-regulated in *rse1* prior to the onset of leaf senescence. We found that SA accumulation was significantly increased in *rse1.* The *rse1* phenotypes are dependent on *SA-INDUCTION DEFICIENT 2* (*SID2*), supporting a role of SA in accelerated leaf senescence in *rse1*. Furthermore, RSE1 protein was localized to the cell wall, implying a possible link between the cell wall and RSE1 function. Together, we show that RSE1 negatively modulates leaf senescence through an *SID2*-dependent SA signaling pathway.

## Introduction

Senescence is the last stage of plant development and is a genetically programmed and evolutionarily advantageous process. Plant senescence occurs at the cellular, tissue, organ, and organismal levels, and leaf senescence has been extensively studied in the past years. Leaf senescence is marked by an enhanced metabolic transition of macromolecules from biosynthesis to degradation, and the nutrients are recycled to sink organs such as young leaves, reproductive organs, and seeds. It has been shown that leaf senescence is modulated by multilayered, multidimensional regulatory mechanisms, including transcriptional and post-transcriptional regulation, protein modification, and hormone signaling (Woo et al., 2013; Schippers, 2015; Yolcu et al., 2018). The mechanism underlying the onset of leaf senescence is elusive, and NAC and WRKY family transcriptional factors have been identified as key regulators acting upstream of leaf senescence onset by turning on senescence-associated genes (SAGs) (Woo et al., 2013). Leaf senescence is also triggered by external stimuli for increasing plant fitness to environmental changes. For instance, hormones, light conditions, salt, and biotic stress-induced senescence involve differential regulation of age-dependent SAGs (Balazadeh et al., 2010; Al-Daoud and Cameron, 2011; Sakuraba et al., 2014; Fernandez-Calvino et al., 2016).

Plant hormones play a crucial role in the regulation of senescence by integrating internal and external signals (Khan et al., 2014). Salicylic acid (SA) is a well-known defense hormone, which plays a critical role in the plant immune response as well as in leaf senescence (Park and Paek, 2007; Vlot et al., 2009). The SA levels increase in senescing leaves, and the mutants for the SA biosynthesis or signaling genes, *PHYTOALEXIN DEFICIENT 4* (*PAD4*) and *NON-EXPRESSER OF PR GENES 1* (*NPR1*), as well as the *NahG* transgenic plants show delayed leaf senescence and reduced transcript levels of several SAGs (Morris et al., 2000). Furthermore, an increased SA accumulation results in early leaf senescence accompanying the up-regulation of age-dependent *SAGs* (Vogelmann et al., 2012; Lee et al., 2016; Li et al., 2016; Huang et al., 2018). Conversely, impaired senescence signaling pathways affect plant defense responses (Carviel et al., 2009; Zhu et al., 2011; Piisila et al., 2015), suggesting possible crosstalk between leaf senescence and the defense mechanisms. Interestingly, recent studies have revealed a link between SA-mediated leaf senescence and the modification of lipids and small molecules by glycosylation, *S*-palmitoylation, and mannosylation (von Saint Paul et al., 2011; Mortimer et al., 2013; Lai et al., 2015; Zhao et al., 2016; Huang et al., 2018). For example, loss-of-function mutations in *UGT76B1* glycosyltransferase (GT) that facilitates the glycosylation of isoleucic acid result in early leaf senescence accompanied with increased levels in SA and an up-regulation of defense-related genes and *SAGs* (von Saint Paul et al., 2011). However, detailed underlying mechanisms remain unclear.

Glycosylation is involved in various cellular processes and plays a vital role in the glycodiversity of macromolecules and the signaling complexity in plants. GTs catalyze the transfer of carbohydrate moieties to a vast range of acceptor molecules including nucleic acids, saccharides, lipids, proteins, and other organic compounds. GTs exist in most living organisms including bacteria, virus, archaea, and eukaryotes, and are classified into over 100 distinct families (GT1–GT107)^1^ (Lombard et al., 2014). Particularly, plant genomes encode highly diverse GT enzymes (Coutinho et al., 2003), reflecting their functional divergence in cellular homeostasis and physiology. For example, the *Arabidopsis* genome encodes 565 GT genes, which consists over 40 families (see text footnote 1). Glycosylation by GTs plays a role in the regulation of plant development, defense, and adaptation (Wang and Hou, 2009).

One of the key functions of GTs is the biosynthesis of polysaccharides, a major component of the cell wall (Lampugnani et al., 2018). Broadly conserved GTs and plant-specific GTs including GT2, GT48, and GT14/14-like gene families have been suggested to partake in this process, and these enzymes are mainly localized in the endomembrane system (Zhou et al., 2009; Hansen et al., 2012; Lao et al., 2014; Culbertson and Zabotina, 2015). A subset of 152 cell wall GTs has been implicated in maintaining distinct cell wall properties of the shoot apical meristem (Yang et al., 2016). Glycosylation is a crucial biological reaction in living organisms, but the physiological role of the majority of GTs remains largely unknown. This may be due to the functional redundancy in the large gene families, substrate diversity, and substrate specificity.

Here, we show that RSE1, a GT-like protein, acts as a negative regulator of *Arabidopsis* leaf senescence in an SA-dependent manner. Transcriptome analysis revealed that genes involved in defense response and SA signaling are highly up-regulated in the *rse1* mutant. Consistent with this, the early leaf senescence phenotype of *rse1* was further enhanced upon external SA treatment, and SA levels were significantly increased in *rse1.* A null mutation in the SA biosynthesis gene *SID2*, but not in *PAD4*, restored the early leaf senescence of *rse1*, implying that RSE1 negatively regulates leaf senescence through the *SID2*-dependent SA biosynthesis pathway. The RSE1 protein is localized preferentially to the cell wall. Overall, our results provide an insight into leaf senescence modulated by the crosstalk between glycosylation and SA signaling.

## Materials and Methods

### Plant Material and Growth Conditions

*Arabidopsis thaliana* ecotype Columbia (Col) and Landsberg (L*er*) were used for all experiments. Plants were grown on soil or 1/2 Murashige and Skoog (MS) salts at 22°C under long day condition (16 h light/8 h dark). The T-DNA insertion line in *RSE1* (*rse1-2*; GK791C05) was obtained from the Nottingham Arabidopsis Stock Centre (NASC). *sid2-1* and *pad4-1* were previously described (Ng et al., 2011). At least five independent transgenic lines were used for each analysis.

Mapping of the *rse1-1* mutation, whole genome sequencing library construction and analysis *rse1-1* was crossed with Col-0 to generate mapping populations. Twenty-four F2 plants exhibiting the early leaf senescence phenotype were used for simple sequence length polymorphism mapping and the mutation was mapped to chromosome 5.

For whole genome sequencing, *rse1-1* was backcrossed to L*er* and 50 F2 plants showing the early leaf senescence phenotype were pooled to extract genomic DNA using the DNeasy plant mini kit (Qiagen). DNA library was prepared according to the illumina Truseq Nano DNA Library prep protocol. For sample library preparation, 0.1 ug of high molecular weight genomic DNA (for a 350 bp insert size) was randomly sheared to yield DNA fragments using the Covaris S2 system. The fragments were blunt ended, phosphorylated, and a single ‘A’ nucleotide was added to the 3′ ends of the fragments in preparation for ligation to an adapter with a single-base ‘T’ overhang. Adapter ligation at both ends of the genomic DNA fragment conferred different sequences at the 5′ and 3′ ends of each strand in the genomic fragment. The ligated DNA was PCR amplified to enrich for fragments with adapters on both ends. The quality of the amplified libraries was verified by capillary electrophoresis (Bioanalyzer, Agilent). The library was clustered on the Illumina cBOT station and sequenced paired end for 101 cycles on the HiSeq 2500 sequencer according to the Illumina cluster and sequencing protocols.

Sequence QC was done through FastqQC 0.11.5^2^ and was mapped to customer genome assembly using bwa 0.7.12 (Li and Durbin, 2009). BAM files were realigned with the Genome Analysis Toolkit 3.5 (GATK) IndelRealigner (McKenna et al., 2010), and the base quality scores were recalibrated using the GATK base quality recalibration tool. Variants were called with GATK’s UnifiedGenotyper tool 3.5. The functional information of the variants was annotated using SnpEff 4.1 (Cingolani et al., 2012).

### Plasmid Construction

In order to generate *RSE1* complementary transgenic plants, the genomic DNA of *RSE1* was amplified by PCR, cloned into pENTR/D-TOPO vector (Invitrogen), and subcloned into Gateway binary vectors by LR recombination (Karimi et al., 2005) (Invitrogen). To generate *RSE1* promoter:GUS lines, an approximately 2.0 kb upstream region from the start codon was amplified by PCR and cloned into the pBI101.2 vector between the *Sal*I and *Xma*I restriction sites. The constructs were then transformed into *Arabidopsis* plants by Agrobacterium-mediated transformation using the floral dipping method (Clough and Bent, 1998). The primers used for plasmid construction are listed in Supplementary Table S1.

### RNA Extraction and Real-Time RT-PCR

For gene expression analysis, total RNA was extracted using plant RNA mini prep kit (ZYMO RESEARCH), and cDNA was synthesized using RNA to cDNA EcoDry Premix (Clontech) according to the manufacturer’s instructions. Quantitative real-time PCR was performed by using the iQ^TM^ SYBR Green Super mix (BIO-RAD) in three technical and biological replicates. Statistical analysis was performed using GraphPad Prism.

The gene-specific primers used for PCR are listed in Supplementary Table S1.

### Assays for Measurement of Symptoms of Leaf Senescence

To analyze leaf senescence phenotypes, the third and fourth rosette leaves of each plant were detached and submerged in a lactophenol-trypan blue solution (0.03% trypan blue, 33% [w/v] lactic acid, 33% water-saturated phenol, and 33% glycerol). Samples were incubated at 99°C for 1 min and at RT for 24 h, and washed in a chloral hydrate solution (2.5 g mL^–1^) (Koch and Slusarenko, 1990). For detection of H_2_O_2_, the detached leaves were immersed in 1 mg/ml DAB solution followed by incubation for 4 h in the dark and then boiled in 100% ethanol for 10 min (Rea et al., 2004). For measurements of chlorophyll, the third and fourth rosette leaves of 20-day-old plants (approximately 150 mg) were collected and ground under liquid N2. For chlorophyll extraction, the powder was dissolved in 80% acetone (v/v) in the dark for 30 min, and centrifuged for 15 min at 4°C. The supernatant was collected, and the extraction procedure was repeated. The chlorophyll content was detected using a spectrophotometer at 663 and 645 nm, and the chlorophyll contents were calculated using the formula (8.02^∗^A663 + 20.21^∗^A645) × volume/weight (Lichtenthaler, 1987). The chlorophyll content in the L*er* was defined as 100%. All experiments were repeated using at least three independent plants.

### SA Measurement

The level of SA was measured as previously described (Segarra et al., 2006; Forcat et al., 2008). Briefly, seedling material (200 mg) was harvested into liquid nitrogen and ground under liquid N_2_. The powder was dissolved with 750 μL MeOH-H_2_O-HOAc (90:9:1, v/v/v) and centrifuged for 1 min at 10,000 rpm. The supernatant was collected, and the extraction procedure was repeated. Pooled supernatants were dried under N_2_ and resuspended in 200 μL 20% MeOH. Samples were analyzed using a liquid chromatography coupled to triple quadrupole mass spectrometry (TSQ Quantum ultra EMR, Thermo Fisher Scientific, San Jose, CA, United States) at the Korea Basic Science Institute (Seoul). The SA standard and samples were separated on the reverse-phase column (RocRM C18, 3.0 mm × 150 mm, 5 um, RESTECK). The mobile phases A (0.1% formic acid in water) and B (0.1% formic acid in acetonitrile) were used. The chromatographic run time was 20 min, and the gradient elution profile was 20% for 2 min, 20–100% for 10 min, 100% for 2 min (phase B), and equilibration for 6 min. The flow rate was 0.2 ml/min, and the sample injection volume was 10 ul. The ESI-MS was operated in the negative ion and selected reaction monitoring (SRM) mode. The mass parameters were a spray voltage of 3000 V, sheath gas pressure of 40 (arbitrary unit), aux gas pressure of 10 (arbitrary unit) and capillary temperature of 300°C. The SRM of SA and IS was acquired using an m/z 137.00 > 93.28, and 299.00 > 137.18 transition at a collision energy of 18%.

### mRNA-Seq Library Construction and Data Analysis

RNA purity was determined by assaying 1 μL of total RNA extract on a NanoDrop8000 spectrophotometer (Thermo Scientific). Total RNA integrity was checked using an Agilent Technologies 2100 Bioanalyzer with an RNA integrity number value. mRNA sequencing libraries were prepared according to manufacturer’s instructions (Illumina Truseq stranded mRNA library prep kit). mRNA was purified and fragmented from total RNA (1 ug) using poly-T oligo-attached magnetic beads and two rounds of purification. Cleaved RNA fragments primed with random hexamers were reverse transcribed into first-strand cDNA using reverse transcriptase, random primers and dUTP in place of dTTP. (The incorporation of dUTP quenches the second strand during amplification because the polymerase does not incorporate past this nucleotide.) A single adenine base was added to these cDNA fragments and was followed by adapter ligation. The products were purified and enriched with PCR to create the final strand-specific cDNA library. The quality of the amplified libraries was verified by capillary electrophoresis (Bioanalyzer, Agilent). After qPCR using SYBR Green PCR Master Mix (Applied Biosystems), the index-tagged libraries were combined in equimolar amounts within a single pool. RNA sequencing was performed using an Illumina NextSeq 500 system following the provided protocols for 1 × 75 sequencing.

Data quality control and preprocessing were performed using FastQC. (v0.11.2)^3^. The Trimmomatic (v0.32.3) (Bolger et al., 2014) was used to trim the adapter sequences and low-quality sequences. Remaining reads were aligned to the *Arabidopsis thaliana* L*er*-0 genome using TopHat (v2.0.9) (Trapnell et al., 2009). The L*er*-0 genome was reconstructed using the *Arabidopsis thaliana* Col-0 genome based on the variant call format file for L*er*-0 obtained from the 1001 Genomes Project website^4^. PCR duplicates were removed using Picard (v1.119)^5^, and ‘MarkDupicates’ and only uniquely mapped reads were counted using HTSeq (v0.6.1) (Anders et al., 2015). The differential expression analysis was performed using DESeq2 (Love et al., 2014) and the *P*-values were determined using the Benjamini–Hochberg method (Benjamini and Hochberg, 1995). Differentially expressed genes (DEGs) were identified as the ones that have the adjusted *P*-value < 0.05.

### Gene Ontology Enrichment

Gene ontology (GO) enrichment analysis was performed using DAVID software^6^. The GO terms with Benjamini *P*-value < 0.05 were selected as the ones enriched by the DEGs used.

### Subcellular Localization

For the analysis of subcellular localization of RSE1, *RSE1* cDNA was subcloned into pB7m34GW destination vectors to generate C-terminal fusion with GFP. The expression constructs were co-transformed into *Arabidopsis* with mCherry-tagged markers for various organelles including the Golgi-localized *Man49* (Saint-Jore-Dupas et al., 2006) and plasma membrane-localized *PIP2* (Ivanov and Harrison, 2014). Endoplasmic reticulum (ER) localization constructs contained the ER retention sequences HDEL (Ivanov and Harrison, 2014). For the plasmolysis experiment, plants were treated with 0.8 M mannitol for 30 min (Truernit et al., 2012). Imaging was performed using an LSM 7 DUO (ZEISS) confocal laser microscopy. The primers used for plasmid construction are listed in Supplementary Table S1.

## Results

### *rse1* Displays an Early Senescence and Cell Death Phenotypes

**FIGURE 1:**
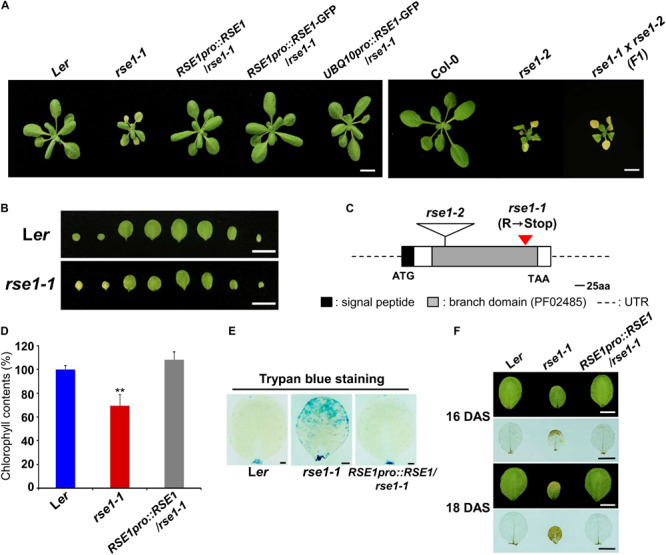
Loss-of-function mutations in *RSE1* cause early senescence and cell death. **(A)** Senescence phenotypes of *rse1-1, rse1-2, RSE1pro:RSE1*/*rse1-1*, *RSE1pro:RSE1*/*rse1-1*, *UBQ10pro:RSE1-GFP*/*rse1-1* at 21 days after sowing, and *rse1-1* × *rse1-2* (F1) plants at 22 days after sowing (DAS). Scale bar, 1 cm. Results are representative of at least five independent plants. **(B)** Detached leaves of L*er* and *rse1-1* at 16 DAS. Scale bar, 1 cm. Results are representative of more than 10 independent plants. **(C)** A schematic diagram of *RSE1* encoding a glucosaminyl transferase (At5g22070) shows the position of the C- to-T mutation in *rse1-1* resulting in a premature stop codon after 300 amino acid and the location of the T-DNA insert in *rse1-2*. **(D)** Relative chlorophyll contents in detached third and fourth leaves of WT, *rse1-1*, and *RSE1pro:RSE1*/*rse1-1* plants at 20 DAS. Error bars indicate SD from three biological replicates. ^∗∗^*p* < 0.01. **(E)** Trypan blue staining of the third leaves of WT, *rse1-1*, and *RSE1pro:RSE1*/*rse1-1* plants at 21 DAS. Scale bar, 0.5 cm. **(F)** 3.3′-Diaminobenzidine (DAB) staining of hydrogen peroxide in the third leaves of WT, *rse1-1*, and *RSE1pro:RSE1*/*rse1-1* plants at 16 and 18 DAS. Leaf yellowing phenotype is observed from 18 DAS in the third leaves of *rse1-1* mutants. Scale bar, 0.5 cm. **(E,F)** Results are representative of three independent plants.

To identify genetic mutants with altered senescence phenotypes, we isolated a mutant, *rapid senescence 1-1* (*rse1-1*) from a genetic screen of EMS-mutagenized plants. Compared with wild-type L*er* plants that retain green and healthy rosette leaves, the cotyledons of *rse1-1* were remarkably yellow, and rosette leaves rapidly turned yellow as they expanded (Figures 1A,B). In contrast to the rosette leaves of WT plants that just began yellowing 3 weeks after sowing, all rosette leaves of *rse1-1* had already turned yellow (Supplementary Figure S1A), indicating enhanced leaf senescence in *rse1-1*. The *rse1-1* mutant also showed other developmental defects, including dwarfism and severely reduced fertility (Supplementary Figure S1C).

To identify the mutated gene, we carried out map-based cloning and whole-genome sequencing in parallel and found a C-to-T mutation in a previously uncharacterized GT-like gene (At5g22070), which introduced a premature termination codon (Figure 1C). The gene product is a member of the GT14/GT14-like family (Ye et al., 2011) and consists of a putative transmembrane domain at the N terminus (1 to 29 amino acid residues) followed by a core-2/1-branching enzymatic domain (72 to 329 residues). The introduction of an *RSE1* genomic fragment (*RSE1pro:RSE1*) and the native promoter/*UBQ10* promoter-driven *GFP*-tagged *RSE1* (*RSE1pro:RSE1-GFP*/*UBQ10pro:RSE1-GFP*) rescued the early leaf senescence phenotype of *rse1-1* (Figure 1A). In addition, a T-DNA insertion mutant in the Col-0 background was obtained from the Nottingham Arabidopsis Stock Center and designated as *rse1-2* (Figures 1A,C). In *rse1-2* mutants, *RSE1* expression was abolished (Supplementary Figure S1B), and leaf senescence was accelerated as in *rse1-1* (Figure 1A). When *rse1-1* was crossed to *rse1-2*, early leaf senescence phenotype was not recovered in F1 plants (Figure 1A), further supporting that functional impairment of *RSE1* results in early leaf senescence.

A typical symptom of leaf senescence is the yellowing induced by chlorophyll degradation. We measured the chlorophyll content in *rse1-1* leaves and found 30% reduction compared with WT and the complemented lines (Figure 1D). Next, we determined cell death and H_2_O_2_ levels using trypan blue and 3,3′-diaminobenzidine (DAB), respectively, because the production of reactive oxygen species (ROS) and the resulting cell death occur during leaf senescence (Koo et al., 2017). We found that both cell death and H_2_O_2_ levels were dramatically increased in *rse1-1* mutants compared with WT and the complemented lines (Figures 1E,F). Furthermore, even when no visible symptoms of senescence were observed in WT, *rse1-1*, and the complemented lines, H_2_O_2_ was detected at the margin of *rse1-1* leaves only (Figure 1F, upper panel). Hydrogen peroxide levels were further intensified and spread to most areas of the leaves in two additional days (Figure 1F, lower panel), suggesting that the enhanced cell death may contribute to leaf senescence in *rse1-1* mutants. Together, these results imply that *RSE1* negatively regulates leaf senescence.

### SAGs Are Up-Regulated in *rse1*

**FIGURE 2:**
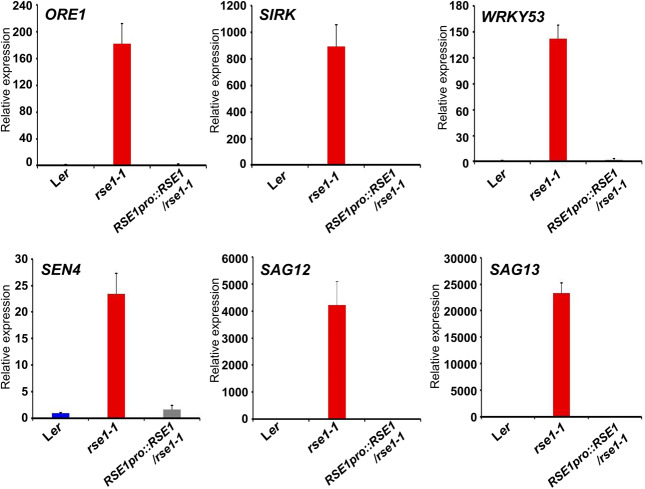
qRT-PCR analyses of senescence-associated genes in *rse1-1.* qRT-PCR indicates a strong up-regulation in expression of the senescence marker genes in *rse1-1*. Third and fourth leaves of WT, *rse1-1*, and *RSE1pro:RSE1*/*rse1-1* plants at 20 DAS were used for RNA extraction and qRT-PCR. The expression levels were normalized to *UBQ5* (At3g62250). The data have been obtained from three biological replicates, and error bars indicate SD.

Previous genetic and transcriptomics studies have shown roles of SAGs and the dynamics of genetic reprogramming in leaf senescence (Hinderhofer and Zentgraf, 2001; Guo and Gan, 2006; Kim et al., 2009; Woo et al., 2016). Transcriptional factors such as NACs and WRKYs also act at early stages of leaf senescence and turn on the downstream genes encoding catabolic enzymes for nutrient recycling (Schippers et al., 2015). We thus analyzed senescence marker genes in the *rse1-1* mutant to examine whether the genetic program governing senescence was affected in *rse1-1*. qRT-PCR analyses showed that the early senescence markers and the genes encoding senescence-induced metabolic enzymes were strongly up-regulated in *rse1-1* compared with WT and the complemented lines (Figure 2). The expression levels of *SAG13* and *SAG12* were increased by about 23,000- and 4,000-fold, respectively, in *rse1-1*. The expression of *SIRK*, which is implicated in immune response, cell death and senescence (Asai et al., 2002; Koo et al., 2017), was increased by 900-fold in *rse1-1*. Furthermore, the central regulator of leaf senescence *ORE1* (Kim et al., 2009) was significantly (180-fold) up-regulated in *rse1-1* compared with WT and the complemented lines (Figure 2). These data suggest that the *rse1* mutation causes accelerated senescence.

### Disruption of *RSE1* Up-Regulates Defense-Related Gene Expression

**FIGURE 3:**
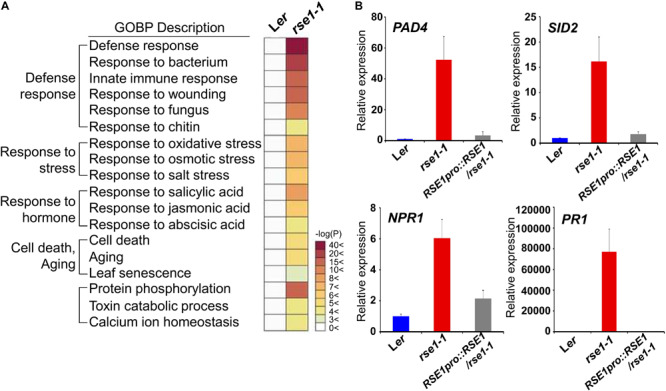
Genes related to defense response are highly up-regulated in *rse1-1.* **(A)** Gene Ontology Biological Processes (GOBP) analysis of differentially expressed genes shows an up-regulation of genes involved in the defense response in *rse1-1*. Third and fourth leaves of WT and *rse1-1* plants at 16 DAS were used for mRNA-seq with three biological replicates. **(B)** qRT-PCR analysis of salicylic acid signaling genes, *PAD4*, *SID2*, *NPR1*, and *PR1* in WT, *rse1-1*, and *RSE1pro:RSE1*/*rse1-1* plants. Third and fourth leaves of WT, *rse1-1*, and *RSE1pro:RSE1*/*rse1-1* plants at 16 DAS were used. The expression levels were normalized to *UBQ5* (At3g62250). The data have been obtained from three biological replicates, and error bars indicate SD.

Next, we conducted transcriptome analysis to further investigate the role of *RSE1*. To focus on the early molecular events that might have caused accelerated leaf senescence in *rse1-1*, leaves of *rse1-1* and WT that showed no visible yellowing were used for analyses. The expression profiling identified 2,186 differentially expressed genes in *rse1-1*. Among these differentially expressed genes, 1,783 were up-regulated, and 403 were down-regulated (Supplementary Tables S2, S3). GO term analysis revealed that the loss-of-function mutation of *RSE1* affected the expression of genes involved in various processes, including aging, stress signaling, and protein phosphorylation (Figure 3A and Supplementary Figure S2). Remarkably, genes involved in defense response were among the highly up-regulated genes in *rse1-1* (Figure 3A). Moreover, genes involved in SA biosynthesis and signaling were also most highly up-regulated in *rse1-1* compared with other hormones (Figure 3A).

Salicylic acid plays an essential role in plant immunity. However, a constitutively high level of SA and activated defense response could be harmful to plants as the plants are forced on alert thereby resulting in stunted plant growth (Seyfferth and Tsuda, 2014). *SALICYLIC ACID INDUCTION DEFICIENT 2* (*SID2*) and *PAD4* regulate SA biosynthesis and accumulation, respectively (Vlot et al., 2009). NPR1 is an SA receptor, which initiates SA signaling (Wu et al., 2012). To validate the up-regulation of genes involved in SA and defense signaling in *rse1-1*, the expression levels of *SID2*, *PAD4*, and *NPR1* were determined by qRT-PCR. All three genes were drastically up-regulated in *rse1-1* compared with WT and the complemented lines (Figure 3B). Furthermore, the level of SA-responsive *PATHOGENESIS-RELATED GENE 1* (*PR1*) was increased by about 80,000-fold (Figure 3B), implying that the disruption of *RSE1* puts the plants on high alert against biotic stress. On the other hand, the expression levels of the early senescence markers such as *ORE1* and *WRKY53* were not changed at the time when defense-related genes were highly up-regulated in *rse1-1* (Supplementary Tables S2, S3). Taken together, these results suggest that SA-mediated defense signaling is associated with enhanced leaf senescence in the *rse1* mutant.

### *RSE1* Is Involved in SA Biosynthesis and Signaling Pathway

**FIGURE 4:**
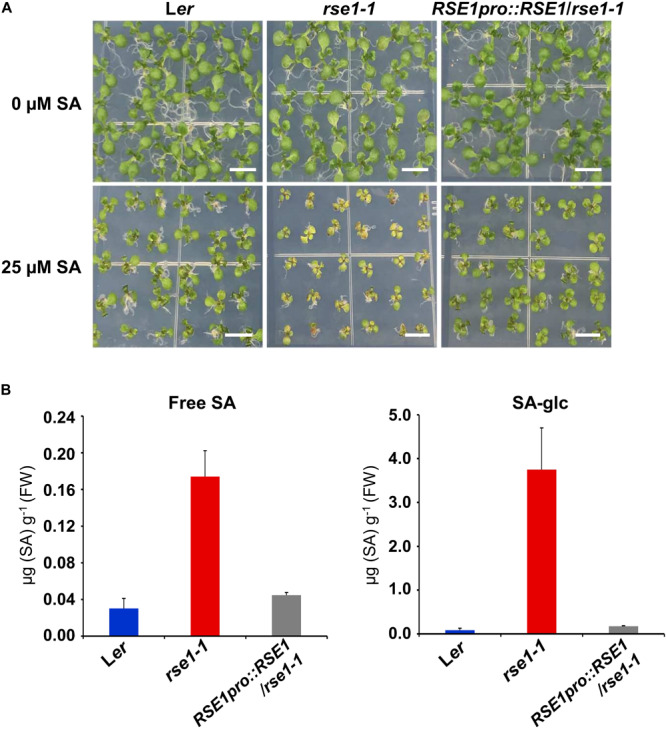
*rse1* accumulates higher levels of SA and is sensitive to SA. **(A)** Exogenous SA inhibits the seedling growth of *rse1-1*. Seedlings were grown on MS media in the absence (0 μM) or presence (25 μM) of SA for 12 days. Results are representative of three independent experiments. Scale bar, 0.5 cm. **(B)** Measurements of free SA and glucose-conjugated SA (SA-glc) in 10-day-old seedlings of WT, *rse1-1*, and *RSE1pro:RSE1*/*rse1-1* grown on 1/2 MS media. The data have been obtained from three biological replicates, and error bars indicate SD.

Since the genes involved in SA signaling were highly up-regulated in *rse1-1* (Figure 3), we tested the involvement of *RSE1* in SA-mediated responses. WT, *rse1-1*, and *rse1-2* plants were grown in the absence and presence of SA to examine whether *rse1* had an altered sensitivity to SA. In the absence of exogenously applied SA, no visible symptoms were observed in WT, *rse1-1*, and the complemented plants. However, *rse1-1* showed retarded growth accompanied by early senescence compared to WT and the complemented plants in the presence of 25 μM SA (Figure 4A). Since *rse1-2* is sterile, the progenies of *rse1-2/*+ heterozygous plants were grown in the absence and presence of SA. Twenty-four out of 141 plants displayed reduced growth and enhanced senescence in the presence of 25 μM SA but not in the absence of SA (Supplementary Figure S3). All the 24 plants turned out to be homozygous for the *rse1-2* mutation, indicating that *rse1* mutants are more sensitive to SA. To determine whether the retarded growth of *rse1-1* was due to the altered level of SA or due to an enhanced sensitivity to SA, we measured contents of free and glucose-conjugated SA (SA-glc) in WT and *rse1-1* mutant plants. It turned out that both free SA and SA-glc levels were increased 6-fold and 45-fold, respectively, in *rse1-1* mutants compared with WT and the complemented lines (Figure 4B). The enhanced sensitivity of *rse1* mutants to SA is probably due to the increased level of endogenous SA in *rse1* mutants, resulting in the increased SA response. Together, these results indicate that *RSE1* negatively regulates SA biosynthesis.

### Early Leaf Senescence of *rse1* Is in Part Attributable to *SID2*

**FIGURE 5:**
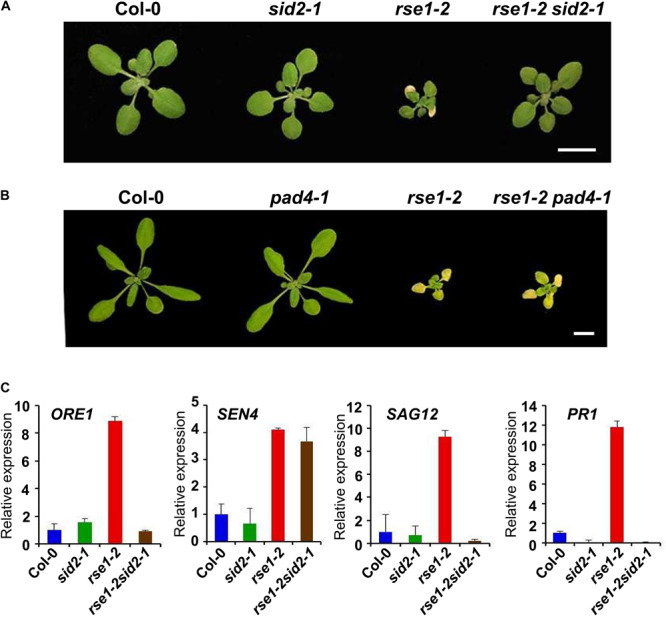
*RSE1* modulates leaf senescence through *SID2*-dependent SA pathway. Phenotypes of WT, *sid2-1*, *rse1-2*, *rse1-2sid2-1* double mutants **(A)** at 17 DAS and WT, *pad4-1*, *rse1-2*, *rse1-2pad4-1* double mutants **(B)** at 22 DAS. Scale bar, 1 cm. Results are representative of at least three independent plants. **(C)** qRT-PCR analyses of senescence- and defense-related genes in WT, *sid2-1*, *rse1-2* and *rse1-2sid2-1* double mutants. Total RNA was extracted from the third and fourth leaves of plants at 20 DAS. The expression levels were normalized to *UBQ5* (At3g62250). The data have been obtained from three biological replicates, and error bars indicate SD.

SID2 and PAD4 are key enzymes in SA biosynthesis and accumulation, respectively (Zhou et al., 1998; Wildermuth et al., 2001). It has been suggested that SID2 and PAD4-dependent SA pathways play a role in leaf senescence (Nawrath and Metraux, 1999; Vogelmann et al., 2012; Guo et al., 2017). Our data suggested that the enhanced SA signaling in *rse1-1* can be attributed to the increased levels of SA and associated with early leaf senescence. To test whether the RSE1 function in leaf senescence depends on the SA signaling pathway, *rse1-2* was crossed to *sid2-1* and *pad4-1* mutants. Interestingly, *rse1-2sid2-1* double mutants largely rescued the early leaf senescence phenotype of *rse1-2* mutants (Figure 5A and Supplementary Figure S4). The size of the plant and developmental defects related to reproduction were also rescued (Supplementary Figure S4B). In contrast, *rse1-2pad4-1* mutants were indistinguishable from the *rse1-2* single mutants (Figure 5B). The phenotypes of the *rse1-2sid2-1* double mutants were in agreement with the expression patterns of the senescence- and SA-related marker genes. The expression levels of *ORE1*, *SAG12*, and *PR1* were reduced to the WT levels in the *rse1-2sid2-1* double mutant (Figure 5C). These results suggest that *SID2* contributes to the early leaf senescence of *rse1*. In contrast, the increased expression of *SEN4* in *rse1-2* was not rescued by *sid2-1*, suggesting that RSE1 may also regulate leaf senescence in a *SID2*-independent manner to a certain extent. The difference in the level of *PR1* expression between *rse1-1* and *rse1-2* is possibly because the basal level of *PR1* in L*er* (*rse1-1* background) at 16 days after sowing was much lower compared to that in Col-0 (*rse1-2* background) at 20 days after sowing, resulting in the smaller fold-change in *rse1-2*. It could be also due to the ecotype difference.

### RSE1 Preferentially Localizes to the Cell Wall

We performed qPCR analysis using leaves, roots, siliques, and flowers to examine the spatial expression patterns of *RSE1* and found that *RSE1* was ubiquitously expressed in the tissues tested (Supplementary Figure S5A). *RSE1* expression patterns were further determined by analyzing transgenic plants harboring an *RSE1*pro:*GUS* construct. Strong GUS expression was detected in the emerging young leaves, and the histochemical GUS staining decreased and was found only in trichomes as the leaves further developed (Supplementary Figure S5B). In roots, GUS expression was strongly detected in root hairs (Supplementary Figure S5B).

**FIGURE 6:**
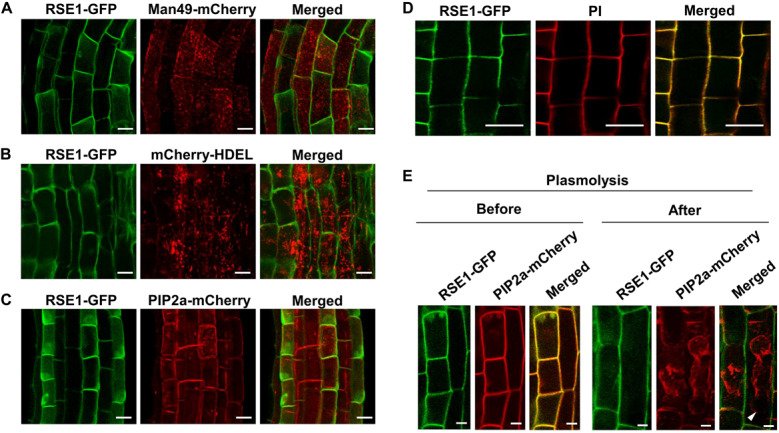
RSE1 is localized to the cell wall. **(A–C)** RSE1-GFP localization in combination with the Golgi localization marker MAN49-mCherry **(A)**, the ER localization marker mCherry-HDEL **(B)**, and the plasma membrane localization marker PIP2a-mCherry **(C)** in 7-day-old seedling roots of *UBQ10pro:RSE1-GFP* plants. Results are representative of at least eight independent transgenic lines. **(D)** *UBQ10pro:RSE1-GFP* transgenic lines were stained with propidium iodide (PI) for cell wall visualization. **(E)** Localization of RSE1-GFP and PIP2a-mCherry before (Mock) and after plasmolysis (0.8 M mannitol) in *RSE1pro:RSE1-GFP* plants. White arrows indicate the plasma membrane of shrunken cells. Scale bar, 20 μm. **(D,E)** Results are representative of at least three independent transgenic lines.

Plant GTs are predicted to mainly localize in the Golgi apparatus and the ER, which most post-translational modifications occur, including glycosylation (Ye et al., 2011; Lao et al., 2014). In order to obtain a clue regarding the function of RSE1, we examined the cellular localization of RSE1 protein by generating transgenic plants expressing a GFP-tagged RSE1 under the control of the native or *UBQ10* promoter. Both the native and *UBQ10* promoter-driven *RSE1-GFP* were able to rescue the early leaf senescence phenotype in *rse1-1* (Figure 1A). We analyzed at least eight independent plants for each construct. Co-localization analyses with the Golgi, ER, and plasma membrane markers in *UBQ10pro:RSE1-GFP* plants showed that RSE1 is not localized to either the Golgi or ER (Figures 6A,B). In addition, DAPI staining revealed that RSE1-GFP is not localized in the nucleus (Supplementary Figure S6A). The RSE1-GFP pattern suggested its localization at the plasma membrane, but the RSE1-GFP signal did not overlap with the plasma membrane marker PIP2a-mCherry and was detected on the outside the plasma membrane (Figure 6C). Optical Z-sections of confocal microscopy show that RSE1-GFP is broadly detected in the cell (top sections) and becomes restricted to the outer layer of the cell (middle sections) (Supplementary Figure S6B). To determine RSE1 localization, we stained the plant cell walls with propidium iodide (PI) and found that RSE1-GFP signals were overlapped entirely with the PI staining (Figure 6D). To further prove RSE1 localization at the cell wall, plant cells expressing both RSE1-GFP driven by the native promoter and PIP2a-mCherry were treated with a high osmolarity solution causing plasmolysis. In contrast to the PIP2a-mCherry that shrank and dislocated, RSE1-GFP was again detected at the cell wall after plasmolysis (Figure 6E). This result implies that RSE1 preferentially localizes to the cell wall.

## Discussion

In this study, we showed that RSE1 negatively regulates leaf senescence through an SA-dependent signaling pathway. Premature leaf senescence is enhanced in *rse1* mutants, accompanying with the up-regulation of senescence marker genes. The phenotypes of the *rse1* mutants, including up-regulation of pathogen-related genes, high accumulations of ROS and SA, and enhanced cell death, are typical defense responses (Jones and Dangl, 2006), which are also common molecular events during leaf senescence. Interestingly, defense signaling is activated prior to the onset of leaf senescence, and SA accumulation is increased in the *rse1* mutant. In addition, early leaf senescence phenotype is rescued by *sid2-1* defective in SA biosynthesis, suggesting that RSE1 may primarily be involved in the defense signaling. In addition to the early leaf senescence phenotype, the *rse1* mutants display severely retarded growth (Supplementary Figures S1A,C). The high level of SA account for the phenotypes because a high dosage of exogenous and endogenous SA causes early leaf senescence and negatively modulates the vegetative growth of plants (Zhang et al., 2007, 2008; Kovacik et al., 2009; Miura et al., 2010; Vogelmann et al., 2012; Lee et al., 2016). Several studies have suggested cross-talk between defense responses and leaf senescence, in which mutants with constant activation of defense signaling display enhanced cell death and leaf senescence (Yoshida et al., 2002; Vogelmann et al., 2012; Lee et al., 2016; Zhang et al., 2017). Together, these findings suggest that RSE1 may primarily act in the SA-dependent defense signaling and cell death, consequently impacting leaf senescence.

RSE1 is previously thought to belong to the Domain of Unknown Function (DUF) 266 proteins. Bioinformatic analyses based on protein sequences and structures have revealed that DUF266 proteins are markedly close to the GT14 family of proteins in the GT superfamilies. Both DUF266 and GT14 proteins encode a branched domain that shows the highest similarity to the core-2/1-branching enzymatic function domain in animals, suggesting that the GT14 and DUF266 family of proteins are closely related to each other (Hansen et al., 2012). GT14 and DUF266 proteins are currently classified into the GT14/GT14-like family (Hansen et al., 2012). Unlike the GT14 proteins, there are high variations in the predicted localization of GT14-like proteins, including the Golgi apparatus, ER, the plasma membrane, extracellular space, and the peroxisomes (Ye et al., 2011). Indeed, the *in vivo* localization of GT14-like proteins remains elusive. For example, rice BC10 containing the DUF 266 domain localizes to the Golgi and regulates cell wall biosynthesis (Zhou et al., 2009). AtGnTL is a GT14-like family protein with the highest similarity to RSE1 among GT14-like proteins in *Arabidopsis* and localizes to the cell wall-associated plasmodesmata (Zalepa-King and Citovsky, 2013). Our data show that RSE1 is localized at the cell wall (Figure 6). This finding suggests that the GT14-like proteins might be involved in more diverse cellular processes due to their assorted localization compared with GTs in other families.

A recent study has revealed that the *Premature Leaf Senescence* (*OsPLS*) gene encoding a putative GT modulates leaf senescence in rice (Ke et al., 2019). OsPLS is a homolog of RSE1, which contains a putative transmembrane domain at the N-terminus and a predicted core-2/1-branching/DUF266 domain (Yang et al., 2017; Ke et al., 2019). *Ospls* mutants show premature leaf senescence accompanied by up-regulated SAGs, elevated ROS levels, and enhanced cell death (Yang et al., 2017; Ke et al., 2019), similar to the phenotypes observed in *rse1*. This indicates that both OsPLS and RSE1 are involved in leaf senescence and cell death. OsPLS regulates leaf senescence and cell death through protein O-glycosylation and ethylene-dependent signaling pathways (Ke et al., 2019). In contrast to *Ospls* in which *O-GlcNAc transferase* (*OGT*) genes *SECRET AGENT1* (*SEC1*), *SEC2* and *SPINDLY* (*SPY*) are significantly reduced (Ke et al., 2019), the expression level of *AtSEC* or *AtSPY* is slightly up-regulated or not altered in *rse1* (Supplementary Tables S2, S3). Moreover, transcriptome analysis and GO term analysis revealed that the ethylene signaling pathway is not nearly affected in *rse1* (Figure 3A; Supplementary Figure S2 and Supplementary Tables S2, S3). RSE1 appeared to modulate SA-mediated signaling thereby regulating leaf senescence and cell death. These studies suggest that molecular mechanisms involving RSE1 and OsPLS may be different, further supporting a functional diversity of the GT14-like genes.

To date, the biological function of GT14/GT14-like family proteins remained very elusive. A few studies have revealed a functional link between GT14/GT14-like proteins and cell wall regulation (Ye et al., 2011). Most of the cell wall biosynthesis-related GT proteins are membrane-associated and are typically localized to the Golgi or the plasma membrane (Ye et al., 2011). RSE1 was also predicted to localize to the Golgi and the plasma membrane. However, our data showed that RSE1 localized to the cell wall (Figure 6). Considering our results and the previous studies, it is plausible to speculate that RSE1 may contribute to the structure and/or function of the cell wall. Additionally, RSE1 contains the plant-specific DUF266 domain (Hansen et al., 2012), thereby raising a possibility that RSE1 may act in a plant-specific process. Furthermore, our transcriptome and Gene Ontology Biological Processes analyses showed that the genes regulating the organization or biogenesis of the cell wall are significantly reduced in *rse1* (Supplementary Figure S2B). Over 130 cell wall-related genes encoding cell wall macromolecule modifying-, cell wall structure regulating-, and secondary cell wall biosynthesis-related proteins are differentially regulated in the *rse1-1* mutants (Supplementary Tables S4, S5). This further implies a role of RSE1 in the regulation of the cell wall. Most sugar nucleotides, donor substrates for GTs, are synthesized in the cytosol and transported to the lumens of the Golgi apparatus and ER by nucleotide sugar transporters for the synthesis of non-cellulosic cell wall polysaccharides (Temple et al., 2016). Since no nucleotide sugar transporters responsible for delivering sugar nucleotides into the cell wall have been identified, we do not exclude the possibility that RSE1 may possess an additional enzymatic activity other than GT. Further biochemical characterization should be able to address RSE1’s enzymatic property.

It is intriguing that the loss-of-function mutation in *RSE1* resulted in the activation of SA-dependent defense signaling. Various lines of emerging evidence have suggested a feedback link between the cell wall function and plant defense mechanisms (Nuhse, 2012). Since plant cell walls are a primary barrier against pathogen attacks, plants turn on defense signaling by monitoring the integrity and function of the cell wall (Nuhse, 2012). For instance, the mutants for POWDERY MILDEW RESISTANT *4* (*PMR4*) encoding a callose synthase and *MURUS 3* (*MUR3*) encoding a xyloglucan galactosyltransferase display cell wall defects, consequently enhanced resistance to pathogens, and increased cell death due to the constitutive activation of SA-dependent defense signaling (Nishimura et al., 2003; Tedman-Jones et al., 2008; Vega-Sanchez et al., 2012). RSE1 localization and the altered gene expression related to the regulatory pathway of the cell wall function in *rse1* indicates a possible role of RSE1 in the regulation of cell wall function. Moreover, defense signaling is highly activated in *rse1*, suggesting a potential link between RSE1-mediated cell wall regulation and defense mechanisms. RSE1, as a putative GT, might directly modify cell wall macromolecules or cell wall proteins that are required for cell wall function or structures. The defects in the cell wall integrity or function caused by the impaired function of RSE1 may be recognized as pathogen attacks, making the plant turn on the constant alert by activating SA-dependent defense signaling, which in turn promotes cell death and subsequent leaf senescence. A further detailed characterization of RSE1 including identification of its molecular targets is noteworthy of investigation.

## Data Availability Statement

The datasets generated for this study can be found in the GenBank, accession no. GSE132740.

## Author Contributions

SL, JK, and YK conceived the study and designed the experiments. SL, M-HK, and JJ performed the experiments. JL analyzed the RNA-seq data. SL, JK, and YK wrote the manuscript.

## Conflict of Interest

The authors declare that the research was conducted in the absence of any commercial or financial relationships that could be construed as a potential conflict of interest.

## Footnotes

1 http://www.cazy.org

2 http://www.bioinformatics.babraham.ac.uk/projects/fastqc/

3 http://www.bioinformatics.babraham.ac.uk/projects/fastqc/

4 https://1001genomes.org/

5 http://broadinstitute.github.io/picard/

6 https://david.ncifcrf.gov/
