# Supplementary material for: Glycosyltransferase-Like RSE1 Negatively Regulates Leaf Senescence Through Salicylic Acid Signaling in Arabidopsis
Source: Front Plant Sci. 2020 May 15;11:551. doi: 10.3389/fpls.2020.00551 (PMC7242760; doi:10.3389/fpls.2020.00551)
Supplement: Supplementary file 2 [file Data_Sheet_2.docx]

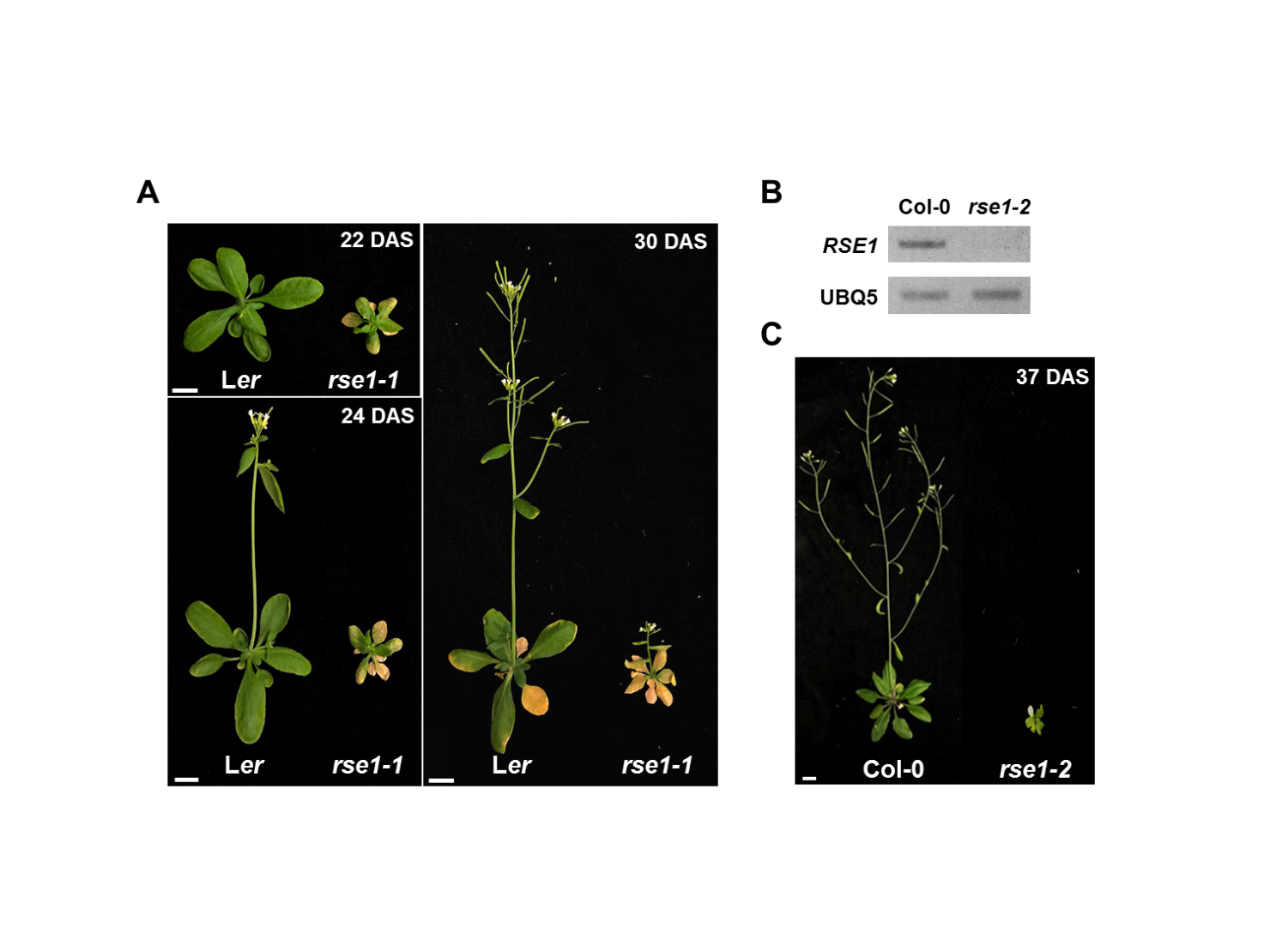


**Supplementary Figure 1. Phenotypes of the *rse1* mutant**.

(A) WT (L*er*) and *rse1-1* plants at 22, 24, and 30 DAS. Scale bars, 1 cm. (B) RT-PCR validates *RSE1* knock-out in *rse1-2*. (C) WT (Col-0) and *rse1-2* plants at 37 DAS. Scale bar, 1 cm. (A, C) Results are representative of more than ten independent plants.


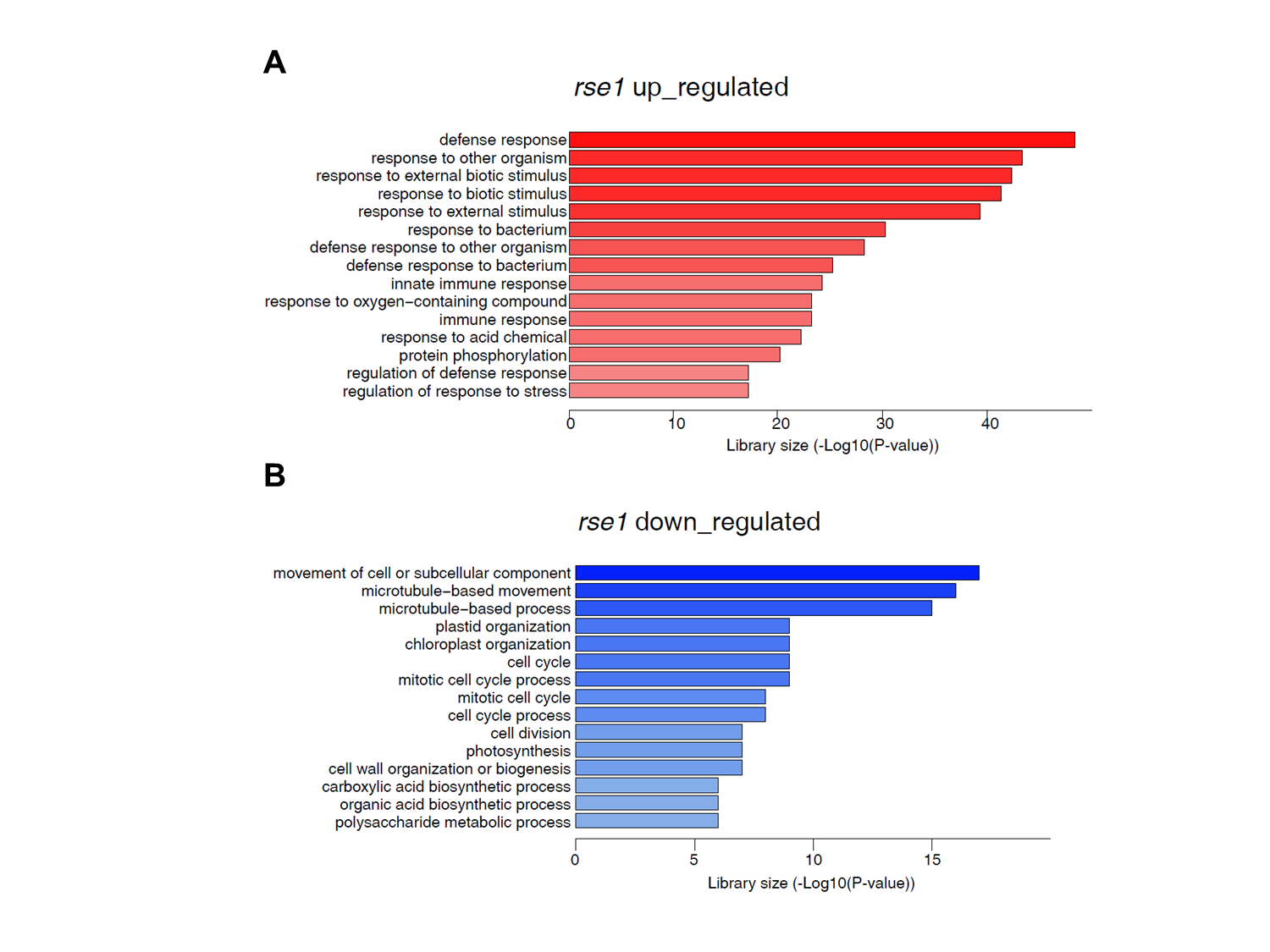


**Supplementary Figure 2. Top 15 GOBP of DEGs up-regulated and down-regulated in *rse1-1*.**

The David tool was used for GOBP enrichment analysis. A Benjamini-Hochberg adjusted p-value (< 0.05) was used as the significance threshold.


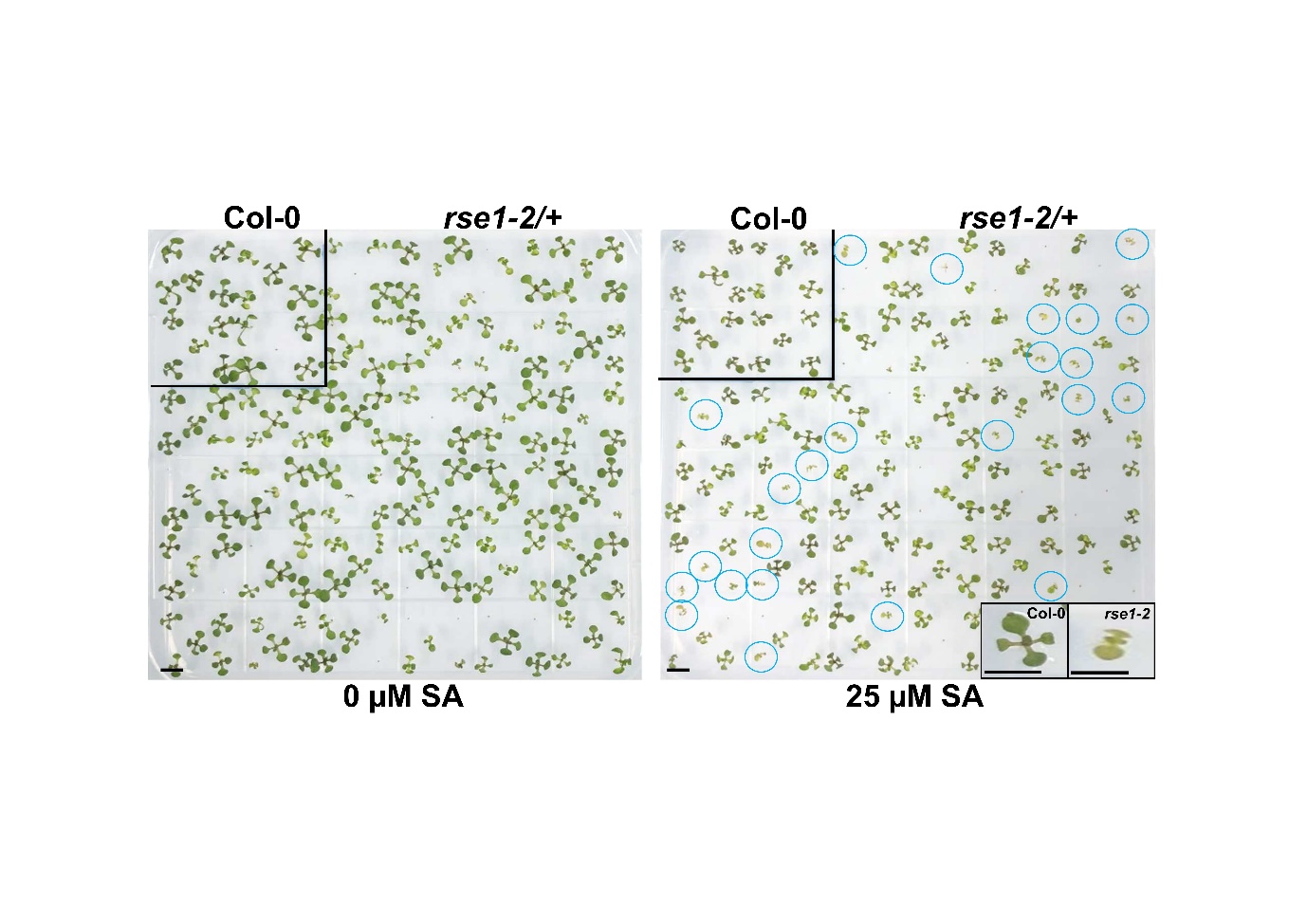


**Supplementary Figure 3. Phenotypes of *rse1-2* in the presence of exogenous SA.**

WT and progenies of *rse1-2/+* heterozygous plants were grown on MS media in the absence (0 µM) and presence (25 µM) of SA for 12 days. Plants showing the premature senescence phenotypes (blue circles) were all homozygous for the *rse1-2* mutation. Insets are blow-up images of representative WT and *rse1-2* seedlings. Scale bar, 0.5 cm.


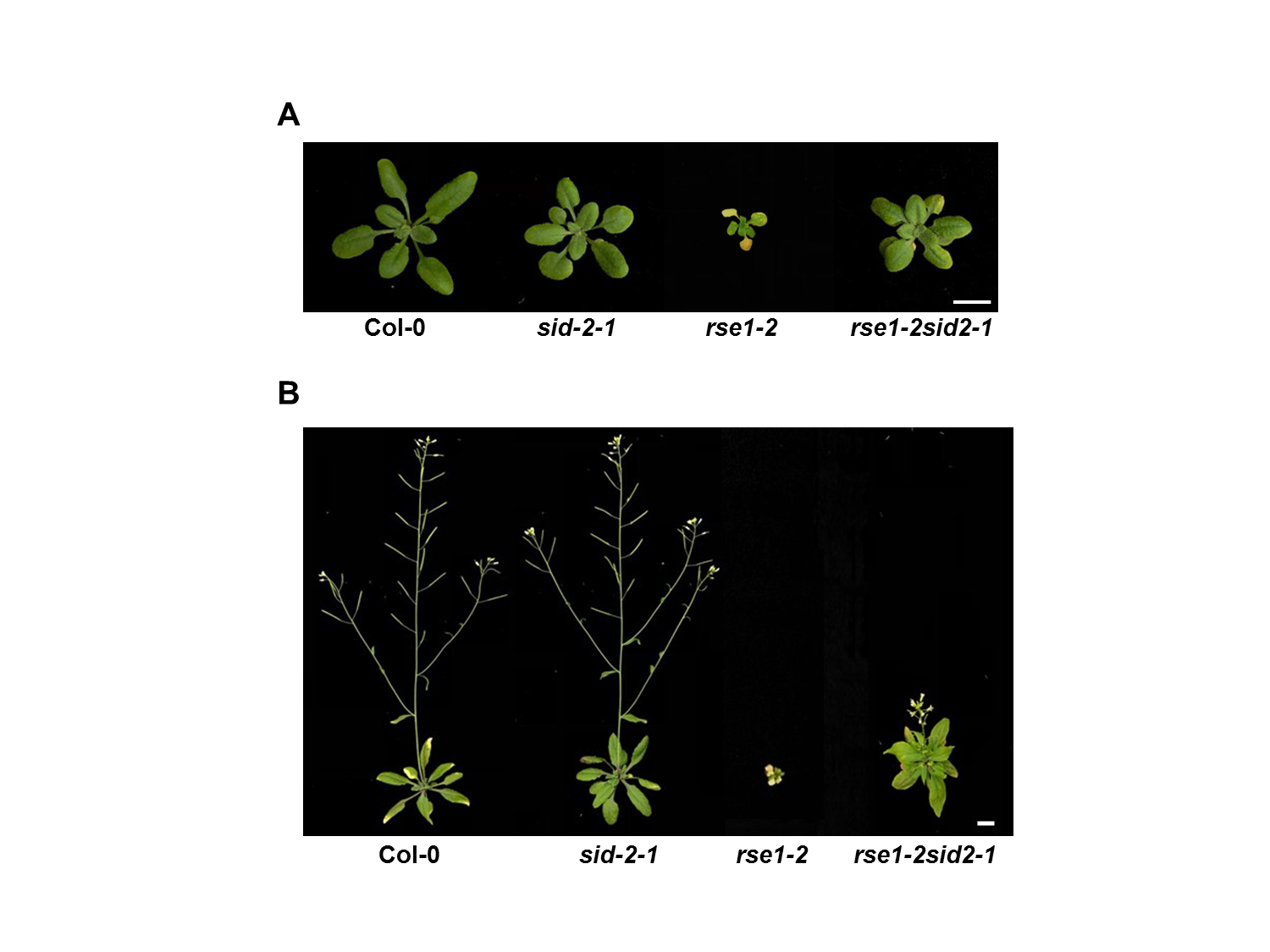


**Supplementary Figure 4. Phenotypes of *rse1-2sid2-*1.**

Phenotypes of WT, *sid2-1*, *rse1-2*, *rse1-2sid2-1* double mutants at 25 DAS (A) and 40 DAS (B). Scale bar, 1 cm. Results are representative of at least three independent plants.


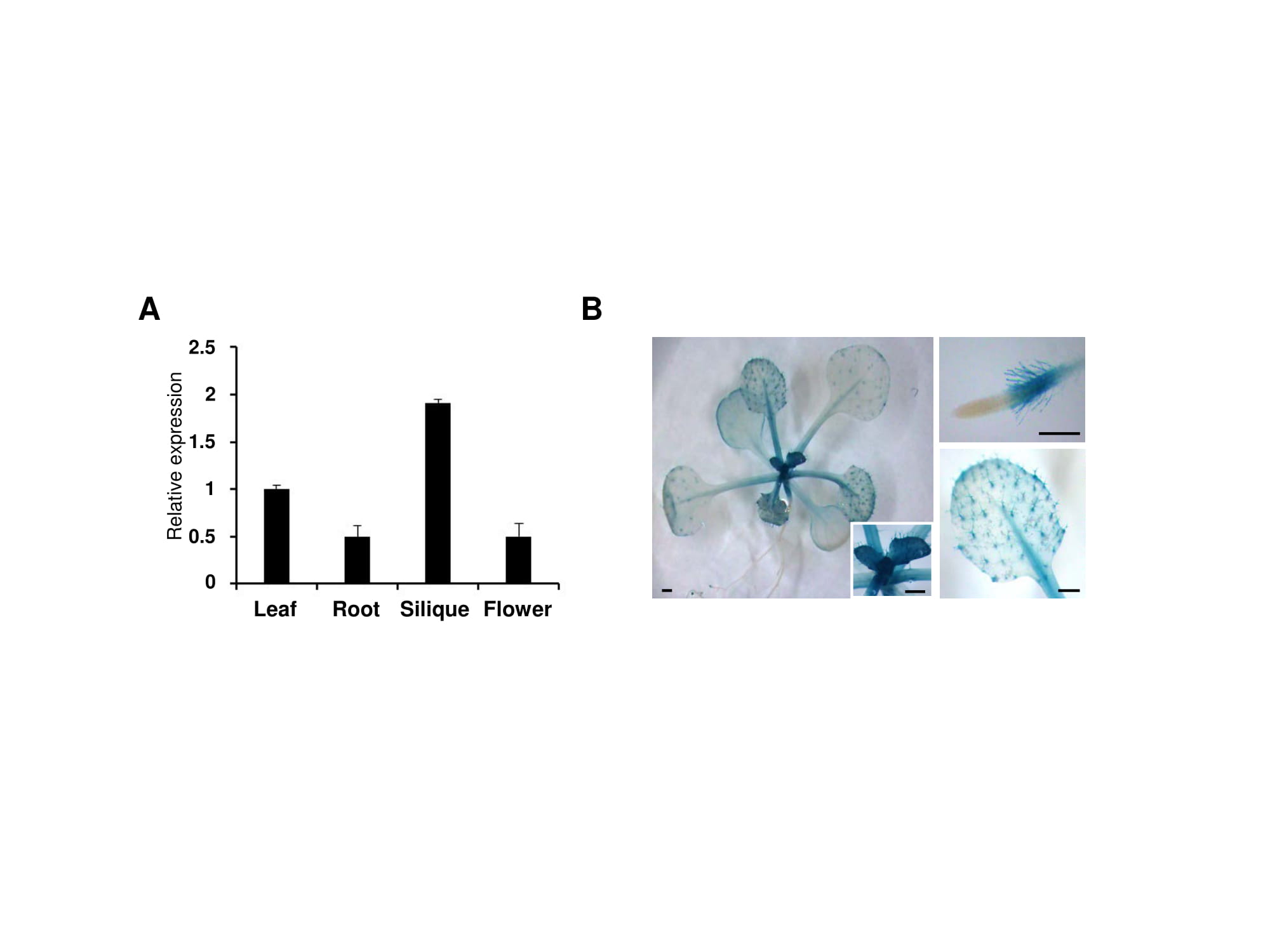


**Supplementary Figure 5. *RSE1* expression in Arabidopsis.**

(A) Expression levels of *RSE1* in various tissues of Arabidopsis at 23 DAS. The transcript level of *RSE1* was analyzed by qRT-PCR and normalized to *UBQ5* (At3g62250). (B) Histochemical GUS staining of transgenic plants carrying a GUS reporter under the control of the *RSE1* promoter. GUS signal was detected in seedlings, trichomes and root hairs. Inset shows young developing leaves at a higher magnification. Plants were stained with 5-bromo-4-chloro-3-indolyl-β-glucuronic acid for 24 h. Scale bar, 0.5 cm. Results are representative of five independent transgenic lines.

**
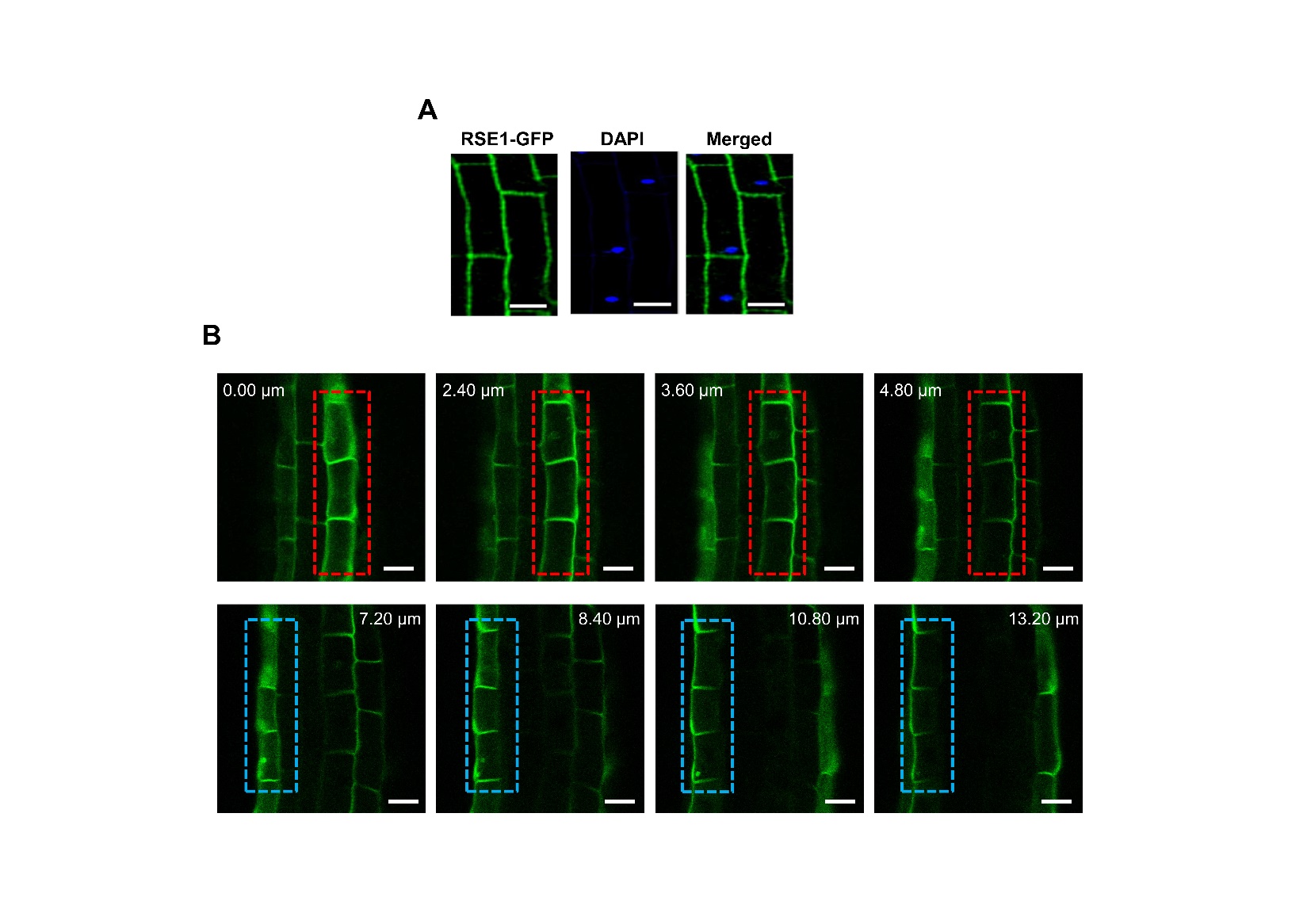
Supplementary Figure 6. Subcellular localization of RSE1.**

(A) *RSE1pro::RSE1-GFP/rse1-1* roots were stained with 4′,6-diamidino-2-phenylindole (DAPI) for nucleus visualization. Blue spots represent the nuclei. Scale bar, 20µm. Results are representative of two independent transgenic plants. (B) Optical Z-section confocal images of root cells of *UBQ10pro::RSE1-GFP*/*rse1-1* plants. Dotted red and blue boxes indicate cells from the top and the middle section images, respectively. Scale bars, 20µm. Results are representative of five independent transgenic lines.
